# Supplementary figures and images for: Immunogenicity Following Two Doses of the BBIBP-CorV Vaccine and a Third Booster Dose with a Viral Vector and mRNA COVID-19 Vaccines against Delta and Omicron Variants in Prime Immunized Adults with Two Doses of the BBIBP-CorV Vaccine
Source: Vaccines (Basel). 2022 Jul 3;10(7):1071. doi: 10.3390/vaccines10071071 (PMC9317843; doi:10.3390/vaccines10071071)

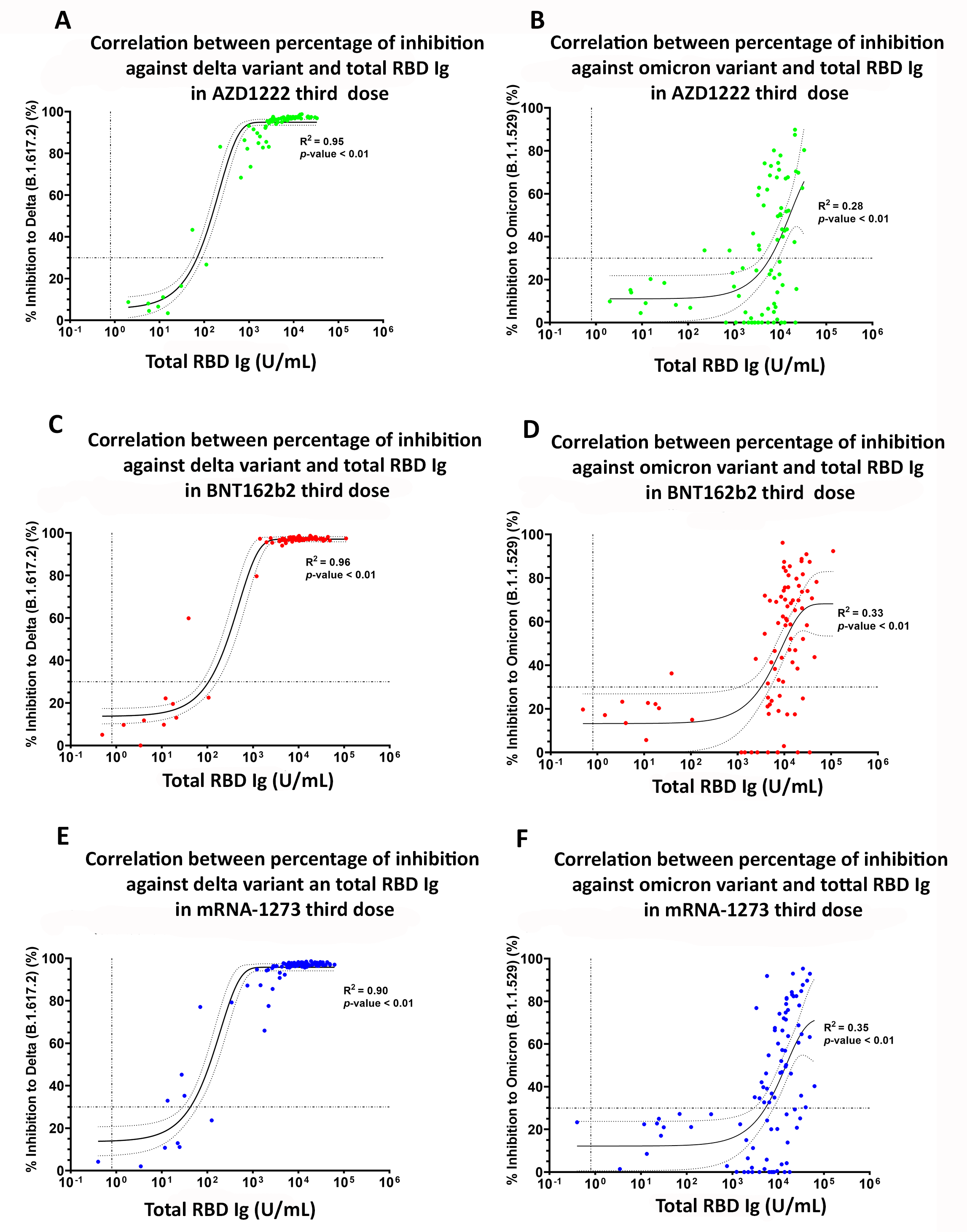

Supplement: Supplementary file 1 [file vaccines-10-01071-s001.zip › Supplement figure S1.tif]

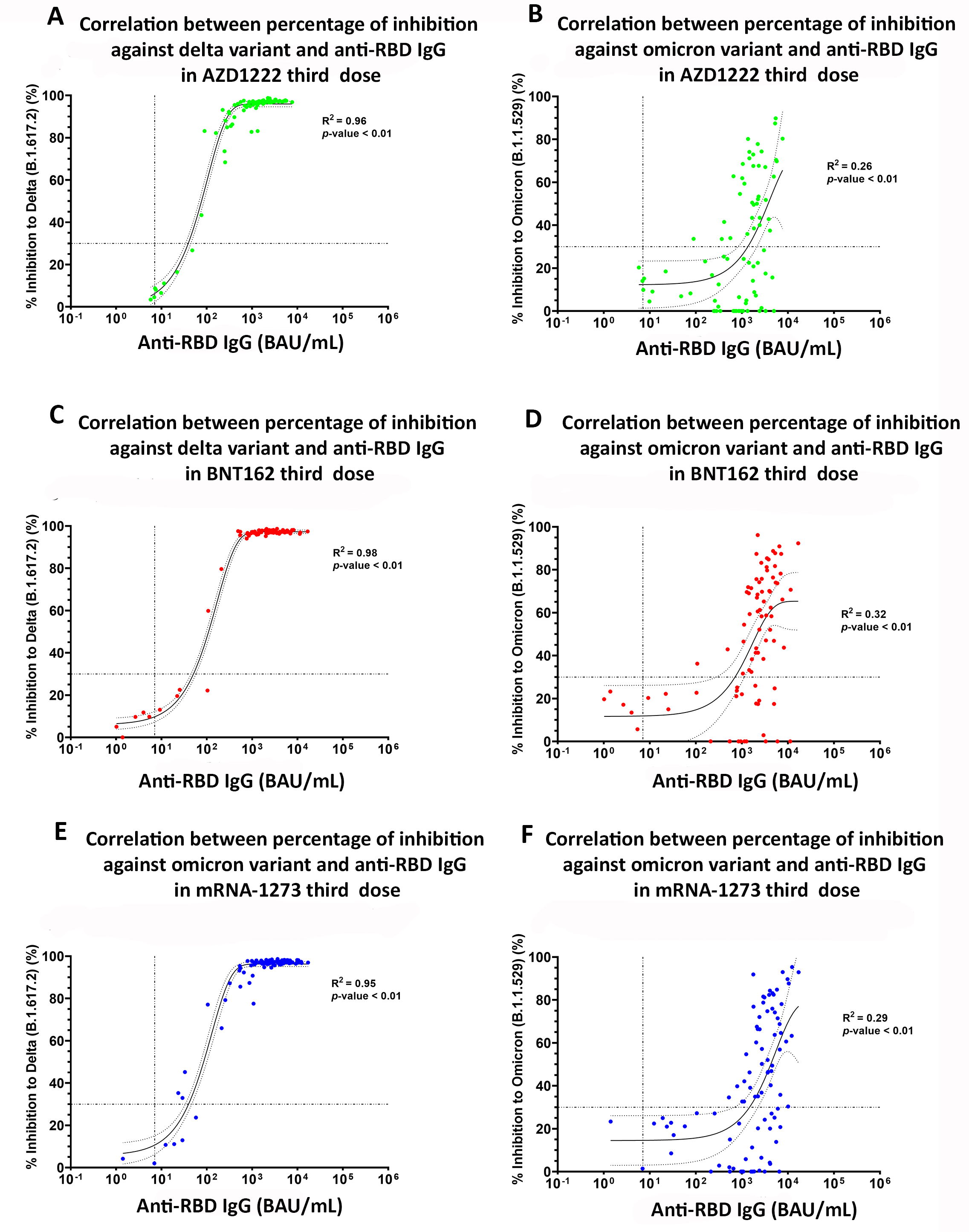

Supplement: Supplementary file 1 [file vaccines-10-01071-s001.zip › Supplement figure S2.tif]
